# Functionalization of Molecularly Imprinted Polymer Microspheres for the Highly Selective Removal of Contaminants from Aqueous Solutions and the Analysis of Food-Grade Fish Samples

**DOI:** 10.3390/polym10101130

**Published:** 2018-10-11

**Authors:** Weixin Liang, Huawen Hu, Wanting Zhong, Min Zhang, Yanfang Ma, Pengran Guo, Meiguo Xin, Mingguang Yu, Haisheng Lin

**Affiliations:** 1 College of Materials Science and Energy Engineering, Foshan University, Foshan 528000, China; lweixin920@126.com (W.L.); huawenhu@126.com (H.H.); zhongwanting1997@163.com (W.Z.); mesyumg@fosu.edu.cn (M.Y.); lhs13763091140@163.com (H.L.); 2 Guangdong Provincial Public Laboratory of Analysis and Testing Technology, China National Analytical Center (Guangzhou), Guangzhou 510070, China; 3 College of Food Science and Engineering, Foshan University, Foshan 528000, China; meiguo@fosu.edu.cn

**Keywords:** molecular imprinting, molecular recognition, emulsion polymerization, MISPE column, stationary phases, adsorption selectivity

## Abstract

The proliferation of pollution in aquatic environments has become a growing concern and calls for the development of novel adsorbents capable of selectively removing notorious and recalcitrant pollutants from these ecosystems. Herein, a general strategy was developed for the synthesis and functionalization of molecularly imprinted polymer microspheres (MIPs) that could be optimized to possess a significant adsorption selectivity to an organic pollutant in aqueous media, in addition to a high adsorption capacity. Considering that the molecular imprinting alone was far from satisfactory to produce a high-performance MIPs-based adsorbent, further structural engineering and surface functionalization were performed in this study. Although the more carboxyl groups on the surfaces of the MIPs enhanced the adsorption rate and capacity toward an organic pollutant through electrostatic interactions, they did not strengthen the adsorption selectivity in a proportional manner. Through a systematic study, the optimized sample exhibiting both impressive selectivity and capacity for the adsorption of the organic pollutant was found to possess a small particle size, a high specific surface area, a large total pore volume, and an appropriate amount of surface carboxyl groups. While the pseudo-second-order kinetic model was found to better describe the process of the adsorption onto the surface of MIPs as compared to the pseudo-first-order kinetic model, neither Langmuir nor Freundlich isothermal model could be used to well fit the isothermal adsorption data. Increased temperature facilitated the adsorption of the organic pollutant onto the MIPs, as an endothermic process. Furthermore, the optimized MIPs were also successfully employed as a stationary phase for the fabrication of a molecularly imprinted solid phase extraction column, with which purchased food-grade fish samples were effectively examined.

## 1. Introduction

Increasing worldwide concern regarding environmental pollution has inspired researchers to explore high-performance functional materials to remediate the contaminated global environment, especially aquatic systems that have a key impact on human life and food supply [1,2,3,4,5,6,7,8,9,10,11,12]. Various kinds of functional materials have thus been reported for achieving environmental remediation [3,4,13,14,15,16,17,18,19,20,21]. Generally, they rely on processes such as photocatalytic degradation, chemical oxidation, membrane separation, chemical precipitation, biological treatment, and adsorption [6,13,14,15,16,17,18,22]. Among these methods relying on functional materials, adsorption processing has been regarded as one of the most viable techniques due to its inherent advantages that include facile operation, low cost, high efficiency, and freedom from the generation of secondary pollutants [23].

Numerous adsorbents have been investigated for the remediation of aquatic environments, such as activated carbon [24], biopolymers [25], graphene [26], textiles [27], and polymer-based hydrogels [28]. However, most of them were employed to deal with a randomly selected aqueous pollutant such as methylene blue (MB) [26] and malachite green (MG) [29], and relatively little attention has been paid to the design and fabrication of adsorbents that exhibit a selectivity for a target organic pollutant among a number of contaminants with similar structural features. This is of great importance if one seeks to detect and analyze a pollutant that is highly toxic toward aquatic organisms and humans. The selective removal of a contaminant not only helps to determine the toxic role that it plays but also reduces the threat that it poses to the environment. Fortunately, molecular imprinting technologies can be employed to afford a high selectivity to an adsorbent for various target species such as enzymes [30], melamine [31], and organic dyes [32]. Molecularly imprinted polymer microspheres (MIPs) are typical examples of products generated by molecular printing [33,34]. However, molecular imprinting alone is insufficient to produce MIPs with satisfactory adsorption selectivity and capacity [35,36,37], and further structural engineering and surface functionalization are required to produce a high-performance MIPs-based adsorbent.

In this study, the microstructure, surface functionalities, and adsorption performance of MIPs were mediated in terms of particle diameters, specific surface areas, pore volumes and surface functionalities by virtue of a combined structural engineering, surface functionalization, and molecular imprinting process. MG was selected as a model organic pollutant and also as the template molecule for the fabrication of the MIPs. MG is considered one of the most hazardous organic dyes, which can pose a long-term toxic, carcinogenic, mutagenic and teratogenic threat to living organisms [38]. The widespread use of MG in the fishing industry readily gives rise to even more disastrous consequences when one considers that fish is such a popular seafood for humans [34]. Herein, a systematic investigation of MIPs used for processing the MG was performed, and their microstructure and surface properties were identified by various characterization analyses. Through structural and property optimizations, excellent adsorption capacity and selectivity of the optimized MIPs toward MG were achieved, far outstripping that of its non-molecularly imprinted counterpart (NIPs). The optimized MIPs were also employed as a stationary phase to fabricate a molecularly imprinted solid-phase extraction (MISPE) column for the facile and continuous processing of MG, with which food-grade fish samples were successfully probed. Therefore, this study could pave the way for the judicious design and preparation of various high-performance adsorbents that possess a unique selectivity toward any target organic pollutant including MG and others.

## 2. Experimental

### 2.1. Preparation of the MIPs

First, Pickering emulsions were prepared using methacrylic acid (MAA) and Ethylene glycol dimethacrylate (EGDMA) as the main precursors. In the synthetic process, the amounts of MAA and EGDMA employed were varied in the ranges of 0.12–0.48 mL and 1.88–1.52 mL, respectively. The EGDMA:MAA (*v*/*v*) feed ratio was changed according to Table 1. Typically, a water phase and an oil phase were separately prepared. The water phase, consisting of MAA (0.12 mL), fumed silica (20 mg), NaOH (3 M, 0.5 mL), Triton X-100 (0.3%, 6 mL), and template (i.e., MG) molecules (20 mg), was homogenized via ultrasonication until the silica was uniformly dispersed in water. Meanwhile, the oil phase, composed of EGDMA (1.728 mL), MG molecules (20 mg), toluene (0.2 mL) and AIBN, was sonicated for 10 min. Subsequently, the water phase and oil phase were homogenized via shaking and then by high-power sonication treatment (500 W). It was found that the higher-power sonication treatment facilitated the formation of smaller liquid droplets. Second, the obtained emulsion was bubbled with N_2_ for 10 min to remove the dissolved O_2_. The emulsion was demonstrated to be stable by observation that no delamination could be noted when it was allowed to stand for 24 h. The free radical polymerization reaction was then triggered by the initiator AIBN in a water bath at 70 °C and subsequently proceeded for 16 h. After the completion of the polymerization reaction, 5 mL of tetrahydrofuran (THF) was added to remove the excess soluble components. The supernatant was removed by centrifugation, and the remaining precipitated microspheres were washed with methanol (MT) and then centrifuged repeatedly until the washing liquid became clear. The silica particles on the surface were removed by soaking in a 30 wt % HF solution for 12 h. Third, the resulting microspheres were extracted under reflux of an MT solution containing 10% acetic acid (by volume) in a Soxhlet extractor until no MG molecules could be detected in the extraction solution. After the removal of the MG molecules, the microspheres were cleaned with a copious amount of MT and then vacuum-dried at 50 °C for 12 h, producing the MIPs-1 sample. The MIPs-2 and MIPs-3 samples were also prepared in a similar manner based on the above procedures and the formulation presented in Table 1. For comparison, the NIPs sample was also fabricated in a similar manner as was employed to prepare MIPs-1 except that the template molecules were not involved.

The other preparation conditions were 20 mg of fumed silica, 0.5 mL of NaOH solution (3 M), 6 mL of 0.3% TX-100, and 200 μL of toluene. Note that, except for the sample NIPs, the preparation of the other samples was involved in the addition of the template molecule MG (20 mg).

### 2.2. Verification of Carboxyl Functionalities by Fluorescent Labeling with Acriflavine

The carboxyl groups on the various prepared MIPs or NIPs samples were labeled with a fluorescent marker acriflavine via a previously reported procedure [2]. Briefly, 10 mg of the prepared sample MIPs-1 was added into 1 mL of an acriflavine solution (100 mg/L in methanol), and the mixture was homogenized at room temperature in the dark for 12 h. The labeled-microspheres were separated by centrifugation, cleaned with excess methanol until no fluorescence could be detected in the supernatant, and dried in a vacuum chamber. These acriflavine-labeled polymer microspheres were deposited onto a glass slide and subsequently observed with a fluorescence analyzer.

### 2.3. Selective Adsorption Tests

A series of MG, MB, basic yellow 1 (BY), and safranine T (ST) standard solutions were prepared in a parallel fashion. The molecular structures of these organic dyes are presented in Figure S1 (Supplementary Materials). To each solution, a deuterated MG solution (10 mg/L, 0.1 mL) was uniformly mixed as the internal standard. Electrospray ionization was employed as the ionization source, and high-performance liquid chromatography (HPLC) coupled with ion trap mass spectrometry (ITMS) was used to measure the mixed solution (MS conditions: 40 psi of atomized gas, 10 L/min of dried gas at 350 °C, and the scanning range of 100–500 *m*/*z* was employed). The concentration ratio of the controlled specimen to the internal standard was adopted as the *x*-coordinate, while the response value ratio was employed as the *y*-coordinate to plot the standard curve.

A batch of the prepared microspheres (30 mg) was placed into different conical flasks, into which a mixed dye solution (25 mL) containing MG, BY, MB, and ST (all of the dye concentrations were fixed to 5 mg/L) was added. The mixture was agitated at a frequency of 120 rpm in a thermostatic water bath at 30 °C for 3 h. After adsorption, the mixed solution (1 mL) was withdrawn into a sampling vial for testing. The parameters, including the distribution coefficient (*K*_d_), selectivity coefficient (*k*’) and relative selectivity coefficient (*K*_0_), were used to evaluate the performance of the MIPs, which can be calculated according to Equations (1)–(3) [39]:

(1) $$K_{d}=\frac{q_{e}}{C_{e}}$$

where *q*_e_ and *C*_e_ represent the equilibrium adsorption capacity and equilibrium mass concentration, respectively.

(2) $$k^{\prime }=\frac{K_{d_{\left(MG\right)}}}{K_{d_{\left(X\right)}}}$$

where *K*_d_(*MG*)__ and *K*_d_(*X*)__ denote the distribution coefficients of MG molecules and competing molecules, respectively.

(3) $$K_{0}=\frac{{k^{\prime }}_{M}}{{k^{\prime }}_{N}}$$

where *k*’_M_ and *k*’_N_ represent the selectivity coefficients of MIPs and NIPs, respectively.

### 2.4. Adsorption Kinetics, Isotherms, and Thermodynamics

MIPs or NIPs (30 mg) were added into a flask (250 mL), into which the 10 mg/L MG solution (100 mL) was mixed. After homogenization, the flask was placed in a thermostatic water bath at 30 °C and shaken at a frequency of 120 rpm. At a given time interval, the solution in the flask was sampled in order to determine the MG concentration based on the as-obtained standard curve (*y* = 0.0598*x* − 0.0304, *R*^2^ = 0.9991), and the amount of the MG molecules that were adsorbed onto the polymer microspheres was calculated as well. Pseudo-first order and pseudo-second-order kinetic models were employed to fit the experimental data. The pseudo-first-order and pseudo-second-order kinetic models can be expressed by Equations (4) and (5), respectively [40]:

(4) $$q_{t}=q_{e}(1-e^{-k_{1}t})$$

(5) $$q_{t}=\frac{t}{\frac{1}{k_{2}{q_{e}}^{2}}+\frac{t}{q_{e}}}$$

where *k*_1_, *k*_2_, *q*_e_, and *q*_t_ denote the pseudo-first order adsorption rate constant (1/min), the pseudo-second-order adsorption rate constant (1/min), the equilibrium adsorption capacity (mg/g) and the adsorption capacity at time *t* (mg/g), respectively.

For determination of isothermal adsorption properties, MIPs or NIPs (10 mg) were placed into a centrifugation tube. Separately, the MG solutions at a series of concentrations (including 40, 50, 60, 70 and 80 mg/L) were prepared, and 3 mL of each MG solution was then added into the centrifugation tube which was subsequently placed into an oscillator (at a frequency of 120 rpm) equipped with a thermostatic water bath that was heated at 35 or 45 °C. The adsorption interactions were allowed to proceed for 3 h, and the concentration of the remaining MG solution was then examined, and the amount of adsorbed MG was determined. Langmuir (Equation (6)) and Freundlich (Equation (7)) isothermal adsorption models were adopted to fit the experimental data, and these models are usually employed to investigate isothermal adsorption processes [41]:

(6) $$q_{e}=\frac{bC_{e}q_{m}}{1+bC_{e}}$$

(7) $$q_{e}=k_{f}{C_{e}}^{n}$$

where *q*_e_, *q*_t_, *C*_e_, *b*, *k* and n represent the equilibrium adsorption capacity (mg/g), the mono-layered and saturated adsorption capacity (mg/g), the equilibrium mass concentration (mg/L), the equilibrium adsorption constant (L/mg), and an arbitrary constant associated with the adsorption intensity, respectively.

For investigation of thermodynamic functions, MIPs or NIPs (0.01 g) were added to a centrifugation tube (5 mL), into which 3 mL of the MG solution (60 mg/L) was mixed. The mixture was then agitated in an oscillator at a frequency of 120 rpm. This oscillator was equipped with a thermostatic water bath that was heated at 30, 40 or 50 °C. After adsorption for 3 h, the residual concentration of the MG solution was measured, and the amount of adsorbed MG was determined. The equations [42,43] used for the calculation of the thermodynamic parameters are presented in the Supplementary Materials (Equations (S1–S4)).

### 2.5. Optimization of the MISPE Conditions

HPLC was employed in the present test to probe MG, and the conditions adopted for the HPLC measurement were provided as follows: (i) a mixed solution of 80% acetonitrile (ACN) and 20% (by volume) aqueous ammonium acetate (abbreviated as AA, with the concentration of 0.125 mol/L and pH 4.5) was employed as the mobile phase, with a flow rate of 1.3 mL/min; (ii) a sampling dosage of 20 μL was used; (iii) a detection wavelength of 618 nm was selected; (iv) a Cnw C18 chromatographic column (250 × 4.6 mm, 5 μm) was employed; and (v) a DAD detector was adopted. A series of MG solutions at different concentrations were prepared, and the standard curve was depicted as *y* = 0.0319*x* − 0.0113, *R*^2^ = 0.9997, by the external standard method [3,4,5].

ACN was employed to prepare different sample solutions on the basis that it has been regarded as the one of the most commonly-used and effective extraction agents [6,7,8] to examine the impact of the solvent used for preparing the MG solution on the adsorption efficiencies of the MISPE column filled with MIPs or NIPs. First, MIPs or NIPs (75 mg) were placed into an empty solid-phase extraction column, equipped with sieve plates at both the top and the bottom of the column (to avoid the falling of the microspheres), thus providing the MISPE column. Second, different sample solutions (including water–ACN mixtures at volume ratios of 0/100, 25/75, 50/50, 75/25, and 100/0) were then used as the solvent to prepare the 50 ng/L MG solution. Third, the MISPE column was activated via the passage of 2 mL of high-purity water and then 2 mL of methanol, prior to the draining of the activated column using a vacuum pump. Fourth, various MG solutions (1 mL) were prepared using different kinds of solvents, and these solutions were then charged into the column and allowed to pass before the column was subsequently drained with a vacuum pump. The residual solution was then collected after it had passed through the column, and the MG concentration within this solution was subsequently measured.

MT, ACN, MT-AA (0.125 M), and ACN-AA solvent mixtures were used as the eluents, and an investigation of their impacts on the elution rate through the MISPE columns filled with MIPs and NIPs was performed. The procedures used to fill and subsequently to activate the column were similar to those described above. Briefly, after the MG solution (50 ng/L) had passed through the MISPE column, the various prepared eluents were allowed to elute through the column containing the adsorbed MG, and the residual solution that eluted out of the column was tested.

### 2.6. Tests on Food-Grade Fish Samples

Fish samples (including basa and cod) were used to evaluate the viability of these MIPs for real-world applications such as the detection of contaminants in food. These fish samples (purchased from Walmart) were minced with a household meat mincing machine and then stored in a plastic box at −18 °C prior to use. Subsequently, a blank fish sample (2.5 g) and the fish samples after processing by the standard (0.02, 0.1, and 0.2 μg/g) were placed into centrifugation tubes (10 mL), and then activated alumina (2.5 g) was added, along with ACN (5 mL) as the extraction agent. The mixture was well homogenized and then sonicated for 20 min, and subsequently centrifuged at 3000 r/min for 10 min. The resulting supernatant was transferred into 10 mL centrifugation tubes, dried under a flow of N_2_, and diluted with water to a volume of 3 mL. This prepared solution (3 mL) was then allowed to pass through the activated MISPE column in three portions. Subsequently, the column was drained using a vacuum pump, and the mixed solution of ACN and AA (0.5 mL) was then added into the drained column in two portions. The solution that remained after the elution was collected for the measurement. The following conditions were employed for the HPLC measurements: a mixed solution of 80% (*v*/*v*) ACN and 20% (*v*/*v*) of aqueous AA (0.125 M, pH 4.5) was employed as the mobile phase, with a flow rate of 1.3 mL/min, and a sampling volume of 50 μL. For MS characterization, the conditions were as follows: 40 psi of atomized gas and 10 L/min of dried gas at 350 °C.

## 3. Results and Discussion

### 3.1. Synthesis of MIPs

**Figure 1:**
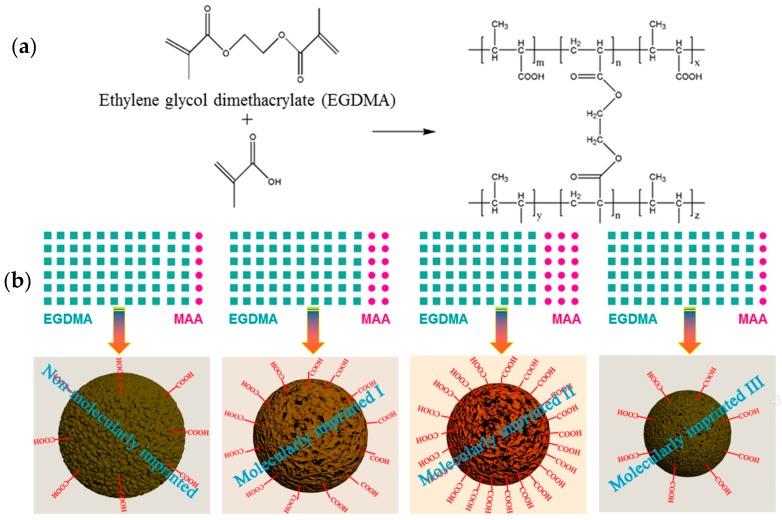
(**a**) Molecular structures illustrating the primary procedure for the synthesis of MIPs based on the free radical polymerization and cross-linking reactions; (**b**) Illustration of the structural features of various polymer microspheres prepared via Pickering emulsion polymerization in combination with molecular imprinting, including particle diameter and surface carboxyl functionalities, as well as the structural variations imparted via the molecular imprinting. The significant parameter (i.e., the EGDMA:MAA ratio) adopted for the synthesis of these polymer microspheres is also illuminated.

The molecular structures and schematic diagrams showing the main reaction are presented in Figure 1a,b. The free radical polymerization of MAA and EGDMA was triggered by the initiator AIBN, leading to the formation of a polymer with a cross-linked network structure which facilitated the adsorption and immobilization of the MG molecules. The molecular imprinting with MG as the template and the subsequent adsorption of MG could then be readily achieved. In addition, ion pairs could be formed between the ionized carboxyl groups of the MAA (i.e., carboxylate) and cationic MG molecules, thus facilitating ionic bonding interactions.

During the synthesis of the MIPs, the addition ratio of the functional monomers to the cross-linking agent played a significant role, as it can directly affect the structure and surface properties of the resulting MIPs (Figure 1b). Table 1 summarizes the experimental observations obtained for various microspheres, as synthesized by the emulsion polymerization of the functional monomer and cross-linking agent at different addition ratios. The samples listed in Table 1 (progressing from top to bottom) were synthesized with increasing amounts of MAA. The samples (denoted as *a* and *b*) were prepared at the EGDMA:MAA addition ratios of 100:0 and 97:3, respectively. The Pickering emulsions at these two ratios exhibited a low stability since the formed emulsion system underwent demulsification reactions under heating conditions. The demulsification caused the formation of a highly cross-linked bulk polymer, rather than the desired polymer microspheres (Figure S2, Supplementary Materials). In contrast, polymer microspheres could be formed in the cases of the other samples including NIPs, MIPs-1, MIPs-2, and MIPs-3 after Pickering emulsion polymerization had proceeded for 16 h. The yields of these emulsion polymerizations are presented in Table 1, along with the average diameters calculated for these prepared polymer microspheres. It was apparent that the produced microspheres were synthesized in a quantitative manner, revealing the success of the polymerization of MAA and EGDMA. It is also noteworthy that the yield of the microspheres was reduced when greater amounts of MAA was added, likely due to ineffective polymerization when the monomer MAA accounted for a too high percentage.

### 3.2. Characterization of the Synthesized MIPs

**Figure 2:**
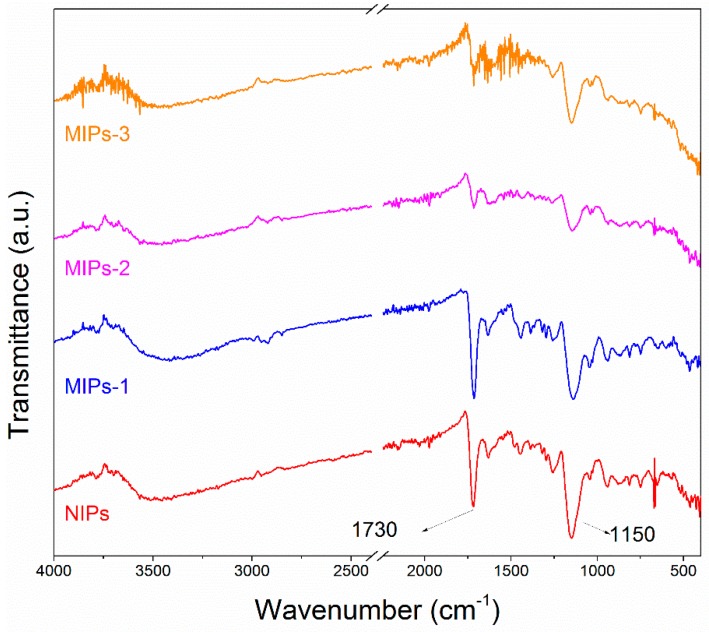
FTIR spectra of the prepared samples: NIPs, MIPs-1, MIPs-2 and MIPs-3.

**Figure 3:**
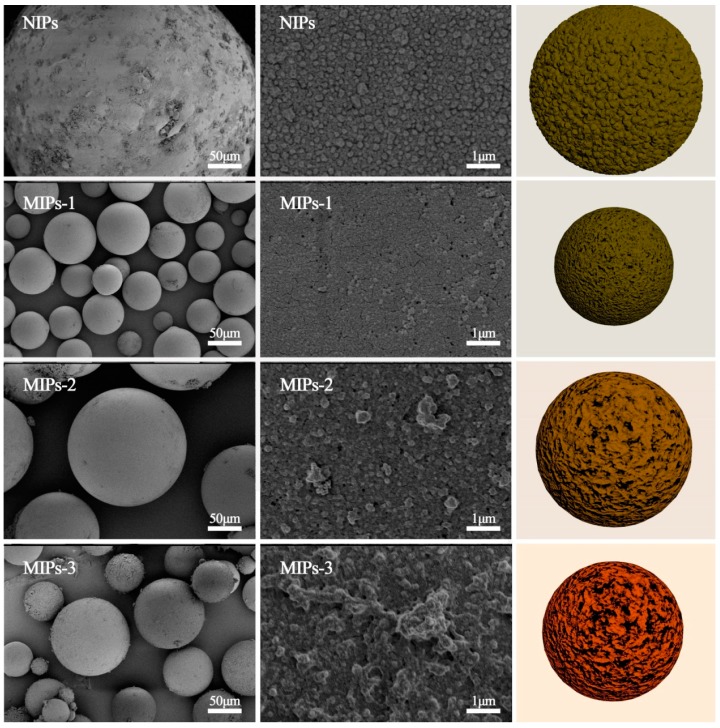
SEM images of the prepared samples including NIPs, MIPs-1, MIPs-2, and MIPs-3, along with schematic depictions of their particle sizes and structural features.

The FTIR spectra of the synthesized MIPs-1, MIPs-2 and MIPs-3, as well as the control sample NIPs, are provided in Figure 2. The absorption at 1730 cm^−1^ can be assigned to the stretching vibration of the carboxyl moieties of the MAA, while the absorption at 1150 cm^−1^ corresponds to the stretching vibration of the C–O–C group present in the formed polymer. It can be noted that all of the prepared samples exhibit nearly identical absorption characteristics, except for the FTIR absorption assigned to the carboxyl functionalities, thus revealing that they had similar structural compositions with a difference in the concentration of carboxyl groups. SEM observations of these samples are shown in Figure 3, along with their schematic depictions. All of the prepared MIPs exhibited a regular spherical shape. The synthetic conditions exerted a significant impact on the surface morphology and porous structure of the resulting polymer microspheres. Cracks and pores with different sizes were observed on the surfaces of the four types of as-prepared polymer microspheres, among which MIPs-1 exhibited more uniform cracks with smaller dimensions. This is most likely to endow MIPs-1 with the best adsorption selectivity for MG. In comparison to the bulk polymerization processes that require ball milling and grinding, Pickering emulsion polymerization has obvious advantages. For example, the spherical surface of the polymer microspheres as synthesized by Pickering emulsion polymerization facilitates the homogeneous distribution of molecular imprinting sites on the surfaces of the MIPs. Without the need for destructive processes such as ball milling, the specific adsorption sites can also be better protected during the Pickering emulsion polymerization process.

Particle diameter distribution histograms of the various synthesized microspheres are illustrated in Figure S3 (Supplementary Materials), and the calculated average diameters of the prepared MIPs are presented in Table 1. The prepared MIPs exhibit a particle diameter range of 19–44 μm. Meanwhile, the sample NIPs that were prepared in the absence of molecular imprinting exhibited a particle diameter (>100 μm) that was obviously larger than that of its molecularly imprinted counterpart MIPs-1 (153 μm vs. 19 μm, see Table 1). This reveals that the template MG molecules played a significant role in stabilizing the emulsion droplets, leading to the formation of more uniform polymer microspheres with a smaller diameter.

**Figure 4:**
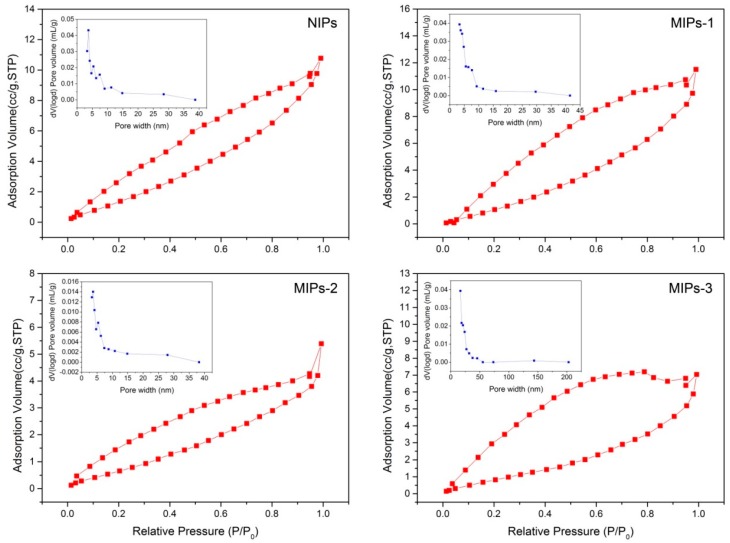
N_2_ adsorption–desorption isotherms for the prepared NIPs, MIPs-1, MIP-2 and MIP-3 samples; insets show the corresponding pore size distributions.

Figure 4 displays the nitrogen adsorption–desorption isotherms as well as the corresponding pore size distribution lines, and the specific results are summarized in Table S1 (Supplementary Materials). Hysteresis phenomena could be observed for all the prepared polymer microspheres (e.g., the adsorption and desorption lines are not overlapped), as a result of the capillary condensation on the porous surfaces of the polymer microspheres. The prepared MIPs-1, MIPs-2, and NIPs samples showed a smaller average pore size ranging from 5 to 50 nm, as compared to the sample MIPs-3 with a mean pore size ranging from 20 to 200 nm, which was consistent with the results obtained via SEM characterization.

**Figure 5:**
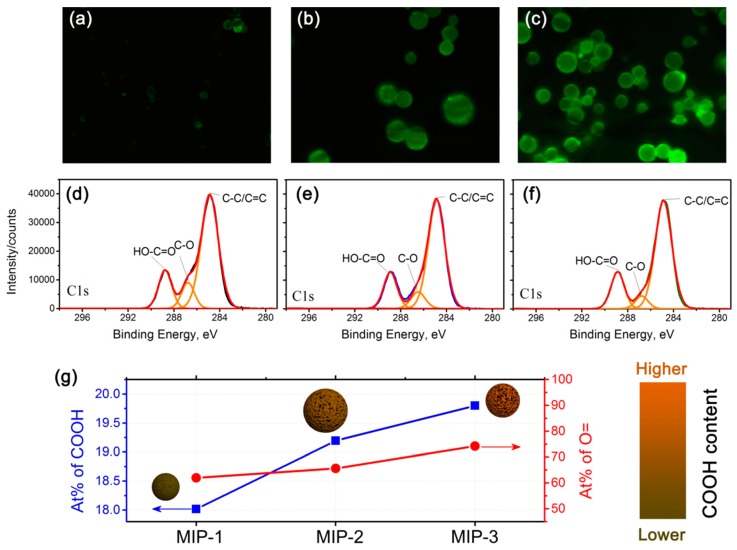
Investigation of the relative content of the carboxyl functionalities in the prepared MIPs-1, MIPs-2, and MIPs-3 samples. (**a**–**c**) Fluorescence images of the prepared: MIPs-1 (**a**); MIPs-2 (**b**); and MIPs-3 (**c**) samples recorded after binding with a florescent dye (in this case acriflavine). (**d**–**f**) High-resolution C1s XPS core-level spectra of the: MIPs-1 (**d**); MIPs-2 (**e**); and MIPs-3 (**f**) samples. (**g**) Comparison plots of the relative atomic percentage of COOH and O= functional groups for the prepared MIPs-1, MIPs-2, and MIPs-3 samples, together with a schematic illustration of their structural features.

Considering that the MAA:EGDMA feed ratio plays a pivotal role in determining the carboxyl functionality content on the surfaces of the MIPs, which is closely related to the adsorption performance, a systematic study on the surface functionalities was conducted, and the results are shown in Figure 5. Figure 5a–c shows the fluorescence microscope images of the acriflavine-labeled MIPs-1, MIPs-2, and MIPs-3, respectively. The fluorescence intensity becomes lowered in a progression from MIPs-1 to MIPs-3, revealing that the density of carboxyl groups is gradually increased. The higher is the density of carboxyl groups, the larger is the number of acriflavine molecules that have been successfully incorporated onto the surfaces of the microspheres as labels, implying that a higher fluorescence intensity can be observed. To further validate the increased concentration of carboxyl groups (progressing from MIPs-1, MIPs-2 to MIPs-3), high-resolution C1s XPS core-level spectra are further provided in Figure 5d–f, respectively, and the corresponding O1s XPS core-level spectra are presented in Figure S4 (Supplementary Materials). Indeed, the signal intensities corresponding to surface carboxyl groups are increased as one progresses from MIPs-1 to MIPs-3, with calculated atomic percentages of approximately 18%, 19.2% and 19.8%, respectively (Figure 5g). Although MIPs-1 possesses the lowest atomic percentage of the surface carboxyl groups, it has the highest specific surface area and the largest total pore volume (Figure 4 and Table S1 (Supplementary Materials)), likely resulting from its small particle diameter.

To prepare high-performance MIPs for the selective and efficient adsorption of MG, many variables need to be considered, including the particle size, number of surface carboxyl groups, specific surface area, and total pore volume, as well as the structural and property variations that are imparted via molecular imprinting. Subsequent sections discuss the adsorption performance of NIPs and MIPs, and the relationship between the structure and adsorption performance is also described.

### 3.3. Evaluation of the Adsorption Selectivity of MIPs toward MG

Three different dyes were used as the competing molecules against MG, and ITMS was used to measure the residual concentration of each dye after the adsorption by MIPs, along with the calculation of the adsorption amount of each dye composition. Prior to the preparation of the mixed dye solution, the standard MS spectra were measured for each dye, thus avoiding the overlap of the ion peaks associated with the different dye molecules. The standard MS spectrum of the mixed dye is provided in Figure S5 (Supplementary Materials). The ion peaks at 283.1, 284.2, 315.1 and 329.2 *m*/*z* can be indexed to MG, BY, MB and ST dyes, respectively. To plot an internal standard curve, a series of mixed dye solutions with different concentrations were prepared, and deuterated MG was employed as the internal standard (334.2 *m*/*z*). The results showing the linear correlation coefficients are presented in Table S2 (Supplementary Materials).

**Figure 6:**
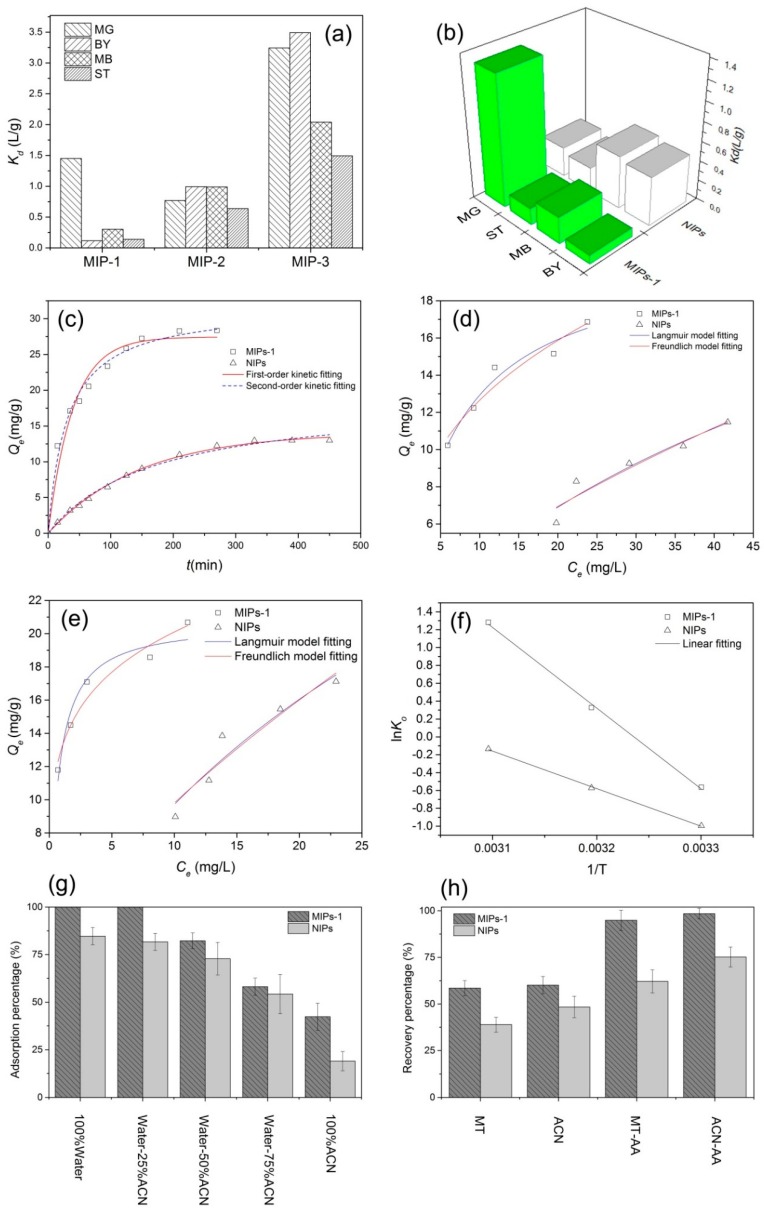
Investigation of the adsorption performance of the prepared MIPs and NIPs samples. (**a**) Comparison of the distribution coefficients (*K*_d_) values of the different competing dyes in a mixed dye solution with MIPs-1, MIPs-2, and MIPs-3 as the adsorbents; (**b**) Comparison of the *K*_d_ values of the different competing dyes in a mixed dye solution using NIPs and MIPs-1 as the adsorbents; (**c**) Experimental data fitted to the pseudo-first and pseudo-second kinetic models as for the adsorption of MG onto the prepared NIPs and MIPs-1 samples; (**d**,**e**) Isothermal adsorption curves for the adsorption of MG onto the NIPs and MIPs-1 at: 303 K (**d**); and 313 K (**e**). (**f**) Plot of ln*K*_0_ as a function of 1/T; (**g**) Comparison of the adsorption percentage obtained with the different solvent composition for the NIPs and MIPs-1 samples; (**h**) Impact of the eluent composition on the recovery percentage for the MISPE columns using NIPs and MIPs-1 as the stationary phases.

Figure 6a and Table 2 present the distribution coefficients of the various prepared MIPs toward different competing molecules. The coefficient is defined as the ratio of the adsorption quantity to the equilibrium mass concentration. A larger distribution coefficient corresponds to a better specific adsorption effect for the given target molecules. Among the molecularly imprinted samples, it is noteworthy that the MIPs-1 sample exhibited the best selective adsorption performance toward MG with a *K*_d_ value as high as 1.45. This distribution coefficient with MG was significantly larger than those of its competing molecules, all of which exhibited corresponding values no larger than 0.3. This indicates that the MIPs-1 sample exhibited significant adsorption selectivity toward MG in the presence of the competing molecules. Thus, this paper focuses on the MIPs-1 sample and its non-molecularly imprinted counterpart (i.e., NIPs) in the following sections.

During the preparation of the MIPs, it was also found that the MAA:EGDMA addition ratio in the emulsion had a great impact on the adsorption selectivity of the resulting MIPs. A higher MAA content could provide the resulting MIPs with higher adsorption capacity, which can be attributed to the incorporation of a greater number of carboxyl on the surfaces of the MIPs, as well as to the formation of large pores (Figure 1, Figure 2, Figure 3, Figure 4 and Figure 5, Table 1, and Table S1 (Supplementary Materials)). The carboxyl groups could form ion pairs with the MG molecules. However, a large MAA:EGDMA ratio yields MIPs with unsatisfactory adsorption selectivity, attributable to the excessive carboxyl groups that are produced which facilitate rapid adsorption at the expense of adsorption selectivity.

The adsorption selectivities of the two parallel samples, namely MIPs-1 (with molecular imprinting) and NIPs (without molecular imprinting), were compared, and the results are shown in Figure 6b and Table 3. The non-molecularly imprinted NIPs sample presents a strikingly different adsorption performance relative to its molecularly imprinted counterpart MIPs-1 (Figure 6b). MIPs-1 possesses a significantly higher *k*’ value (Table 3), verifying the superior adsorption selectivity of MIPs-1 to that of NIPs and hence confirming the success of the present molecular imprinting process.

### 3.4. Analysis of the Adsorption Kinetics

The MG solution and the adsorbent were placed into a conical flask to conduct the adsorption experiment, and the processed MG solution was withdrawn at a given time interval. In Figure 6c, the overall process involving the adsorption of MG molecules onto MIPs-1 includes stages involving rapid and slow adsorption. The adsorption proceeds rapidly within the first 100 min, which becomes slow during the subsequent stage and reaches equilibrium at 200 min, with a gradually lowered adsorption rate and a saturated adsorption level. Such a rapid overall adsorption process can be attributed to the small size of the polymer microspheres as well as their abundance of carboxyl groups. The initial rapid adsorption period is a result of the diffusion of MG molecules through the solution and their subsequent adsorption onto the active sites of the polymer microspheres. In comparison to the NIPs, MIPs-1 showed a higher adsorption rate. Meanwhile, MIPs-1 exhibited a superior equilibrium adsorption capacity (28.36 mg/g) to that of NIPs (12.97 mg/g), revealing the better adsorption performance of MIPs-1 as a result of the smaller particle diameter, larger specific surface area, and superior adsorption selectivity. The data fitting provides us with more specific results, as shown in Table 4. Despite the high correlation coefficients exhibited by the NIPs (exceeding 0.99 for both kinetic models), MIPs-1 obeyed a better fitting to the pseudo-second-order kinetic model. The difference in the fitting of the kinetic models is most likely caused by the specific binding sites-induced chemical adsorption on the surfaces of the MIPs-1.

### 3.5. Isothermal Adsorption Behavior

Figure 6d,e depicts the isothermal adsorption lines for the adsorption of MG molecules onto NIPs and MIPs-1 at different temperatures. It can be found that the adsorption capacity of MG is increased with an elevated initial MG concentration. Under the same adsorption conditions, MIPs-1 exhibited a superior adsorption capacity to NIPs at both 303 and 313 K, being 1.5- and 1.25-fold larger, respectively. At the same temperature, the equilibrium constant of MIPs-1 is also markedly larger than that of NIPs, which can be attributed to the higher specific surface area of MIPs-1 and its higher adsorption selectivity for MG. Table 5 also provides the isothermal absorption parameters for the adsorption of MG onto the microspheres. With an increase in the temperature, the equilibrium adsorption constants (*k*_l_ and *k*_f_) are noticeably increased. Larger equilibrium adsorption constants increase the adsorption power, indicating the adsorption capacity is enhanced at elevated temperatures. Higher temperatures facilitate the adsorption effect, which is most likely due to the fact that the higher temperatures promote the diffusion of the molecules and hence facilitate the adsorption process.

It is interesting to find that neither of the isothermal adsorption models adequately describes the present adsorption of MG molecules onto MIPs-1 and NIPs, due to a low fitting correlation coefficient (below 0.99). This suggests that the adsorption process involved here is more complex than monolayer adsorption alone. Multiple interactions could co-exist in driving the present adsorption process, including the electrostatic, van der Waals and hydrogen bonding interactions [44,45,46,47].

### 3.6. Adsorption Thermodynamics

The plots of the ln*K*_0_ as a function of the reciprocal of the temperature (i.e., 1/*T*) were depicted in Figure 6f, with the specific results summarized in Table 6. The slope and *y*-intercept were calculated as the entropy change △*S* and enthalpy change △*H* for the adsorption systems at different temperatures, respectively. For both MIPs-1 and NIPs adsorbents, the values of △*H* are estimated to be positive, and the value of △*G* is lowered when the temperature increases. This reveals that an increase in the temperature facilitates the adsorption of MG onto both MIPs-1 and NIPs, as the desorption of water molecules from the surfaces of the polymer microspheres requires energy. The values of the total entropy change △*S* for the adsorption of MG onto MIPs-1 and NIPs are both positive, thus confirming that the adsorption system becomes increasingly disordered after the adsorption has taken place. Actually, the accumulation of various molecules including the adsorbate and others would normally cause the system to become more ordered, thereby lowering the entropy. The actual increase in the entropy of the present system can be attributed to a layer of water molecules that are readily adsorbed onto the polymer microspheres prior to the adsorption of the MG molecules. This implies that our adsorption system is a simultaneous process involving the desorption of the previously adsorbed water molecules and the adsorption of the MG molecules. Considering that the molecular weight of a water molecule (18 g/mol) is much lower than that of an MG molecule (365 g/mol), the number of the desorbed water molecules can greatly exceed that of the corresponding number of MG molecules, resulting in the increased disorder of the present adsorption system. We also consider that the MG molecules could also be hydrated, and, before the effective interactions between MG molecules and the surface of MIPs, the hydration waters would need to be displaced; this might also result in the increase in the entropy.

### 3.7. Optimization of the MISPE Conditions

It was found that acetonitrile (ACN) exhibited an excellent extraction capability toward MG in solution, and thus a solvent mixture of ACN and water at different mixing ratios (*v*/*v*) were examined as the eluents. After the MISPE process, the concentration of the residual MG was measured to calculate the adsorption rate, and the measurements were repeated twice, with the results shown in Figure 6g and Table 7. Due to the strong elution power of ACN (as discussed below), the adsorption rate of the MG molecules onto the stationary phase of the MISPE column was gradually lowered as the ACN concentration was increased. No MG could be detected in the residual solutions collected after the MISPE process had taken place when the pure water or the aqueous solvent mixture containing a low ratio of ACN (25% by volume) was employed, and the adsorption rate of the MISPE column reached its maximum value. It is noteworthy that, irrespective of the eluent composition, higher retention capacities for MG were achieved when MIPs-1 served as the stationary phase rather than non-molecularly imprinted NIPs sample, which can be attributed to the higher adsorption selectivity imparted via molecular imprinting, as well as to the more dense packing resulting from the smaller diameter of the MIPs-1.

Before the practical measurements were performed, the MG molecules that were retained by the MISPE had to be removed via elution to avoid false positive results. High-purity water was used as the sample solvent for MG, and a series of eluents were then adopted as mobile phases for the MISPE column. The MG concentrations in the eluted liquids were determined, along with the recovery rates (Figure 6h and Table 8). It was demonstrated that ACN exhibited a higher elution performance in comparison with MT, and the addition of a weakly acidic buffer salt solution into the pure organic solvent dramatically enhanced the elution capacity. Consequently, a mixed solution of ACN and ammonium acetate (AA) was employed as the eluent.

### 3.8. Analysis of the Food-Grade Fish Samples

MG solutions at various concentrations, including 20, 50, 200 and 400 mg/mL, were passed through a MISPE column filled with MIPs-1. After the elution had taken place with an ACN solution of AA employed as the eluent, the concentration of the MG in the eluted solution was probed by HPLC-MS. The standard chromatogram is displayed in Figure S6 (Supplementary Materials), and the standard curve obtained for the MG solution was measured as *y* = 0.0687*x* − 0.5093, *R*² = 0.9983. It was also found that the linear fitting could be achieved for the standard solutions after they had passed through the MISPE column, which thus demonstrates that the MISPE columns provide a viable means for the analysis of actual samples. The experimental data presented in Table 9 show that neither of the purchased food-grade fish samples (i.e., basa and cod) was contaminated by MG.

## 4. Conclusions

This paper has described the structural engineering and surface functionalization of MIPs for enhancing their ability to selectively remove a target organic pollutant (MG) from aqueous media. In addition, the impact of different parameters employed during the molecular imprinting process on the structures and properties of the resultant MIPs was also investigated. Various preparation conditions were considered to optimize the structure and adsorption performance of the MIPs, such as sonication conditions and the feed ratio between the initial functional monomer MAA and the cross-linking agent EGDMA. The resulting optimized MIPs, with a superior adsorption selectivity and capacity toward MG, were evidenced to possess an appropriate amount of carboxyl groups, a small particle diameter, a large specific surface area, and effective molecular imprinting sites. The MAA:EGDMA ratio in the emulsion exerted a significant impact on the synthesis and selective adsorption performance of the MIPs. For instance, a low MAA ratio could induce demulsification during the synthesis, while an excess of MAA weakened the adsorption selectivity for MG. The optimized particle diameter of the MIPs was found in the range of 7–31 μm, corresponding to a high specific surface area, and the optimized sample, MIPs-1, showed a much higher selectivity coefficient *K*’ value as compared to its non-imprinted counterpart NIPs. The adsorption of the MG molecules in the aqueous solution obeyed a pseudo-second-order kinetic model. The study on the isothermal and thermodynamic equations revealed that higher temperatures facilitated the adsorption of MG onto the MIPs that was an endothermic process. The optimized sample MIPs-1 was also applied as a stationary phase to fabricate a MISPE column, with which the purchased food-grade fish samples including basa and cod were effectively probed.

## Figures and Tables

**Table 1:** Preparation of a variety of MIPs realized by variation of the EGDMA:MAA ratios (by volume) in the formulation, together with the calculation of the average diameters of the prepared MIPs.

Sample Code	EGDMA (mL)	MAA (mL)	Ratio (*v*/*v*)	Experimental Phenomena	Yield (%)	Average Diameter of MIPs (μm) ± SD
*a*	2.00	0.00	100:0	Polymerized into a bulk form	-	-
*b*	1.94	0.06	97:3	Polymerized into a bulk form	-	-
MIPs-1	1.88	0.12	94:6	Uniform fine green particles	98.3	19 ± 5
MIPs-2	1.73	0.27	88:12	Uniform fine green particles	93.4	44 ± 13
MIPs-3	1.52	0.48	76:24	Uniform fine green particles	88.6	22 ± 8
NIPs	1.88	0.12	94:6	Uniform fine gray particles	98.2	153 ± 39

**Table 2:** The selective recognition coefficients of the prepared different MIPs.

Substrate	MIPs-1		MIPs-2		MIPs-3	
	*K*_d_	*k*’	*K*_d_	*k*’	*K*_d_	*k*’
MG	1.45	-	0.77	-	3.24	-
BY	0.12	12.47	0.99	0.77	3.49	0.93
MB	0.30	4.77	0.99	0.78	2.04	1.59
ST	0.14	10.36	0.64	1.21	1.49	2.17

**Table 3:** The selective recognition coefficients of MIPs-1 and NIPs for the MG adsorption.

	MIPs-1			NIPs			
	*C*_e_	*K*_d_	*k*’	*C*_e_	*K*_d_	*k*’	*K*_0_
MG	1.93	1.32	-	3.67	0.30	-	-
BY	4.51	0.09	14.67	3.14	0.50	0.61	23.96
MB	3.78	0.27	4.88	3.04	0.54	0.57	8.61
ST	4.16	0.17	7.82	3.85	0.25	1.21	6.44

**Table 4:** Kinetic parameters obtained for the adsorption of MG by MIPs-1 and NIPs.

AdsorptionSystem		Pseudo-First Order Kinetic Equation			Pseudo-Second Order Kinetic Equation		
	*q*_e,exp_	*q*_e_/(mg/g)	*K*/(1/min)	*R* ^2^	*q*_e_/(mg/g)	*K*/(1/min)	*R* ^2^
MIPs-1 + MG	28.36	27.14	27 × 10^−2^	0.941	31.74	1.03 × 10^−3^	0.993
NIPs + MG	12.97	14.07	6.87 × 10^−3^	0.997	19.07	3.03 × 10^−4^	0.992

**Table 5:** Isothermal adsorption fitting for the adsorption of MG onto NIPs and MIPs-1.

	Langmuir Fitting				Freundlich Fitting		
	*T* (K)	*k*_l_ (L/mg)	*q*_m_ (mg/g)	*R* ^2^	*k*_f_ (L/mg)	*n*	*R* ^2^
MIP-1	303	0.165	20.71	0.960	5.96	0.326	0.942
	313	1.644	20.72	0.946	13.11	0.185	0.962
NIP-1	303	0.015	29.00	0.919	0.88	0.687	0.912
	313	0.026	46.23	0.926	1.92	0.707	0.914

**Table 6:** Thermodynamic parameters obtained for the adsorption of MG onto the NIPs and MIPs-1.

	△*S*/(J/mol)	△*H*/(kJ/mol)	△*G* (KJ/mol)		
			300 (K)	310 (K)	320 (K)
NIPs + MG	106.92	34.93	2.47	1.41	0.34
MIPs + MG	242.85	75.06	1.40	−0.81	−3.18

**Table 7:** Comparison of the adsorption percentages of solid phase extraction columns utilizing NIPs and MIPs as the stationary phase, achieved with different sample solvents.

Sample Solvent	NIPs	MIPs-1
Water (100%)	84.6 ± 4.6%	100.0 ± 0%
ACN (25%, *v*/*v*)	81.7 ± 4.4%	100.0 ± 0%
ACN (50%, *v*/*v*)	72.8 ± 8.5%	82.2 ± 4.1%
ACN (75%, *v*/*v*)	54.2 ± 10.3%	58.1 ± 4.5%
ACN (100%)	18.9 ± 5.0%	42.3 ± 7.1%

**Table 8:** Comparison of the percentages of MG recovered from MISPE columns filled with NIPs and MIPs with the use of different eluents.

Sample Solvent	NIPs-1	MIPs-1
MT	38.9 ± 3.9%	60.1 ±4.1%
ACN	48.4 ± 5.7%	58.5 ± 4.6%
MT-AA	62.1 ± 6.1%	94.9 ±5.4%
ACN-AA	75.2 ± 5.3%	98.4 ± 2.9%

**Table 9:** The test results and standard recovery rate of MG for purchased food-grade fish samples determined after passage through a MISPE column.

Purchased Food-Grade Fish Sample	Added Amount of MG (μg/g)	Concentration of MG in the Eluent (μg/mL)	Mean Recovery Rate (%)	RSD (%)
Basa	0	n.d	—	—
	0.02	0.012	69.9	10.8
	0.1	0.073	87.6	4.6
	0.2	0.142	85.3	4.3
Cod	0	n.d	—	—
	0.02	0.011	64.2	6.5
	0.1	0.074	89.0	4.5
	0.2	0.141	84.5	2.7

Note: n.d. represents no data collected.

## Supplementary Materials

The following are available online at http://www.mdpi.com/2073-4360/10/10/1130/s1. Figure S1: Molecular structures of the organic dyes adopted in this study, including MG and its competing molecules, namely BY, MB and ST, Figure S2: Digital images of the various MIPs synthesized at different volume ratios of the functional monomer to the cross-linking agent, Figure S3: Particle size distribution histograms for the prepared NIPs, MIPs-1, MIPs-2 and MIPs-3 samples, Figure S4: (a-c) High-resolution O1s XPS core-level spectra of the MIPs-1 (a), MIPs-2 (b) and MIPs-3 (c) samples, Figure S5: Mass spectrum of various competing molecules in the mixed dye solution, Figure S6: Standard Chromatogram of a MG solution (0.2 mg/L), Table S1: Summary of the results from the N_2_ adsorption-desorption measurements for the prepared NIPs, MIPs-1, MIPs-2 and MIPs-3 samples, Table S2: Linear correlation coefficients obtained for the competing molecules involved in this study. Supplemental experimental section that includes materials, and characterizations; equations used for the calculation of the thermodynamic parameters; molecular structures of the organic dyes adopted in this study, including MG, MB, ST and BY; digital images of the various MIPs synthesized at different EGDMA:MAA volume ratios; particle size distribution histograms for various prepared samples; high-resolution O1s XPS core-level spectra; a mass spectrum of various competing molecules in the mixed dye solution; a standard chromatogram of a MG solution; and additional tables used to summarize the experiment, characterization and adsorption test results.
