# Supplementary material for: Functionalization of Molecularly Imprinted Polymer Microspheres for the Highly Selective Removal of Contaminants from Aqueous Solutions and the Analysis of Food-Grade Fish Samples
Source: Polymers (Basel). 2018 Oct 11;10(10):1130. doi: 10.3390/polym10101130 (PMC6403773; doi:10.3390/polym10101130)
Supplement: Supplementary file 1 [file polymers-10-01130-s001.pdf]

# Functionalization of Molecularly Imprinted Polymer Microspheres for the Highly Selective Removal of Contaminants from Aqueous Solutions and the Analysis of Food-Grade Fish Samples

**Weixin Liang [1,2,†], Huawen Hu [1,†], Wanting Zhong [1], Min Zhang [1,*], Yanfang Ma [2,*], Pengran Guo [2,*], Meiguo Xin [3], Mingguang Yu [1] and Haisheng Lin [1]**

[1] College of Materials Science and Energy Engineering, Foshan University, Foshan, Guangdong 528000, China; lweixin920@126.com (W.L.); huawenhu@126.com (H.H.); zhongwanting1997@163.com (W.Z.); mesyumg@fosu.edu.cn (M.Y.); 13763091140@qq.com (H.L.)

[2] Guangdong Provincial Public Laboratory of Analysis and Testing Technology, China National Analytical Center (Guangzhou), Guangzhou 510070, China

[3] College of Food Science and Engineering, Foshan University, Foshan, Guangdong 528000, China; meiguo@fosu.edu.cn

* Correspondence: zhangmin@gic.ac.cn (M.Z.); myfedwin@126.com (Y.M.); prguo@fenxi.com.cn (P.G.)

† These authors contributed equally to this work.

**Materials**

Fumed silica (Aerosil 200) of 99% purity was supplied from Evonik Degussa Co. Ltd. Azobisisobutyronitrile (99% purity, AIBN) and methacrylic acid (MAA, 99% purity) were purchased from Tianjin Fuchen Chemical Reagent Factory (Tianjin, China). Triton-100 (Tx-100, 99% purity) was obtained from Sangon Biological Engineering Co., Ltd. (Shanghai, China). Ethylene glycol dimethacrylate (EGDMA, 98% purity) was provided by Aladdin Reagent Co., Ltd. (Shanghai, China). Malachite green (MG), basic yellow 1 (BY), safranine T (ST), and methylene blue (MB) were AR grade and obtained from Tianjin Tianxin Fine Chemical Reagent Center (Tianjin, China). Deuterated MG (99.8%) was purchased from Witega Co., Ltd. Acetonitrile (ACN) and methanol (MT) were both of chromatographic grade and supplied by Tianjin Siyou Fine Chemicals Co., Ltd (Tianjin, China). The fish samples (basa and cod) were purchased from Walmart Stores (Guangzhou, China). All of the other chemicals were AR grade and used as received unless indicated otherwise.

**Characterization**

The surface morphologies of the prepared polymer microspheres were observed using a scanning electron microscope (SEM, Hitachi S4800, Japan). A solid phase extraction device was obtained from Dongkang Sci.-Tech. Co., Ltd (Tianjin, China). The fluorescence images were captured using a three-dimensional fluorescence analyzer (Aqualog, Horiba, Japan). A solution of a fluorescent dye, in this case acriflavine, was prepared via dissolution of the dye powder (10 mg) into methanol (100 mL), which was then stored in a refrigerator for the subsequent fluorescent labeling and imaging. The Brunauer–Emmett–Teller (BET) specific surface area measurements of the prepared NIPs, MIPs-1, MIPs-2 and MIPs-3 samples were performed using a Micromeritics ASAP 2020 nitrogen adsorption apparatus (Norcross, GA, USA). Prior to the BET test, the samples were degassed at 150 °C, and the corresponding pore size distribution was analyzed using the desorption branch by means of the Barrett–Joyner–Halenda (BJH) method [1]. Fourier transform infrared (FTIR) spectra were recorded on an IRAffinity-1S spectrophotometer (Shimadzu, Japan). The mass spectrometric analysis was conducted using a 6540 UHD mass spectrometer (Agilent Technologies, Santa Clara, USA). Ultraviolet–visible (UV/Vis) spectra were recorded with a UV-3100 UV/Vis spectrometer (Shimadzu, Japan).

**The equations used for the calculation of the thermodynamic parameters**

These thermodynamic functions can be calculated according to Eqs. (1-4) [2]:

$$K_0 = \frac{q_e}{C_e} \tag{S1}$$

$$\Delta G = -RT\ln K_0 \tag{S2}$$

$$RT\ln K_0 = T\Delta S^\circ - \Delta H^\circ \tag{S3}$$

$$\ln K_0 = -\frac{\Delta H^\circ}{R}\frac{1}{T} + \frac{\Delta S^\circ}{R} \tag{S4}$$

where $q_e$, $C_e$, $R$, $\Delta H$ and $\Delta S$ denote the equilibrium adsorption capacity (mg/g), equilibrium mass concentration (mg/L), gas constant ($R$ = 8.314 J/mol·K [3]), the enthalpy change (kJ/mol) and the entropy change (J/molK), respectively.

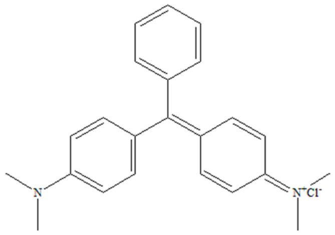

malachite green (MG)

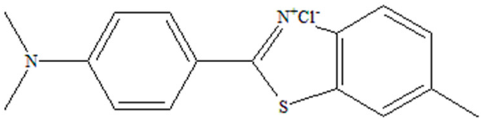

basic yellow (BY)

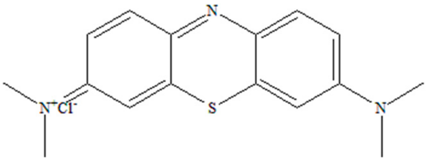

methylene blue (MB)

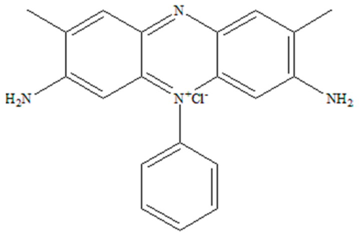

safranine T (ST)

**Figure S1** Molecular structures of the organic dyes adopted in this study, including MG and its competing molecules, namely BY, MB and ST.

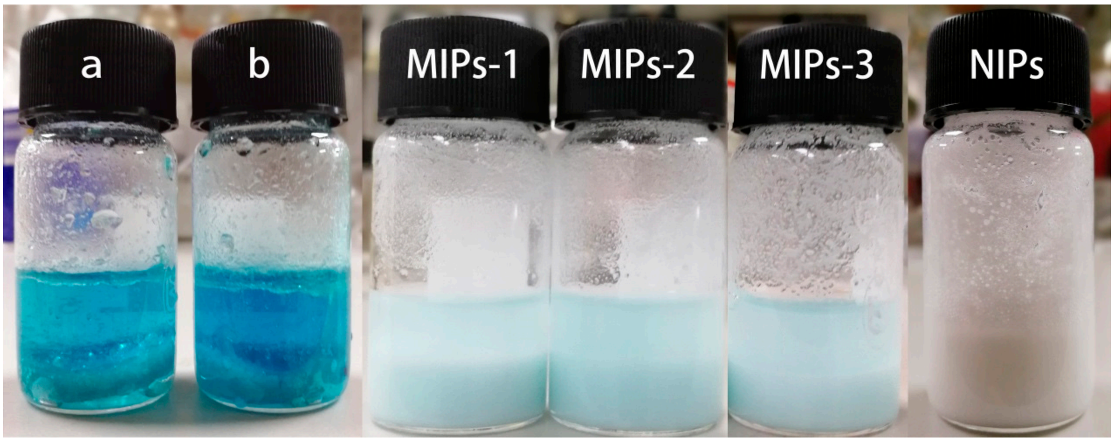


**Figure S2** Digital images of the various MIPs synthesized at different volume ratios of the functional monomer to the cross-linking agent.

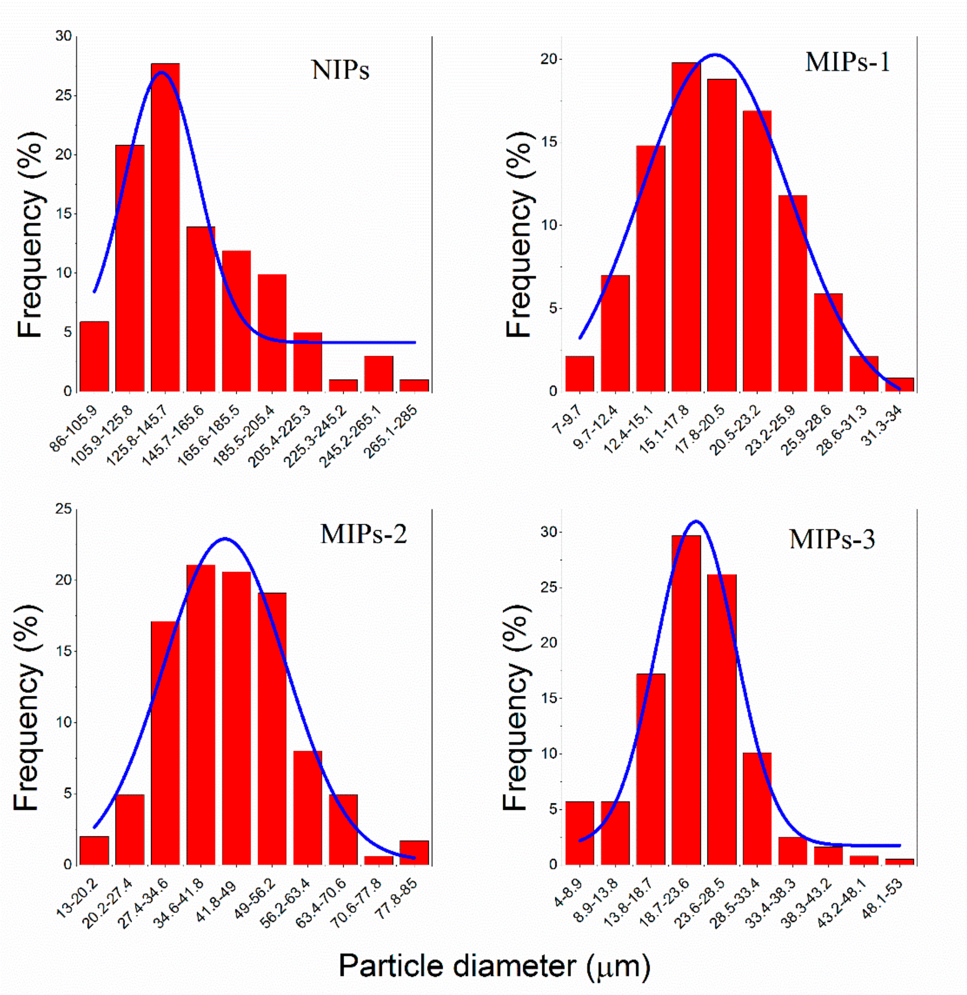


**Figure S3.** Particle size distribution histograms for the prepared NIPs, MIPs-1, MIPs-2 and MIPs-3 samples.

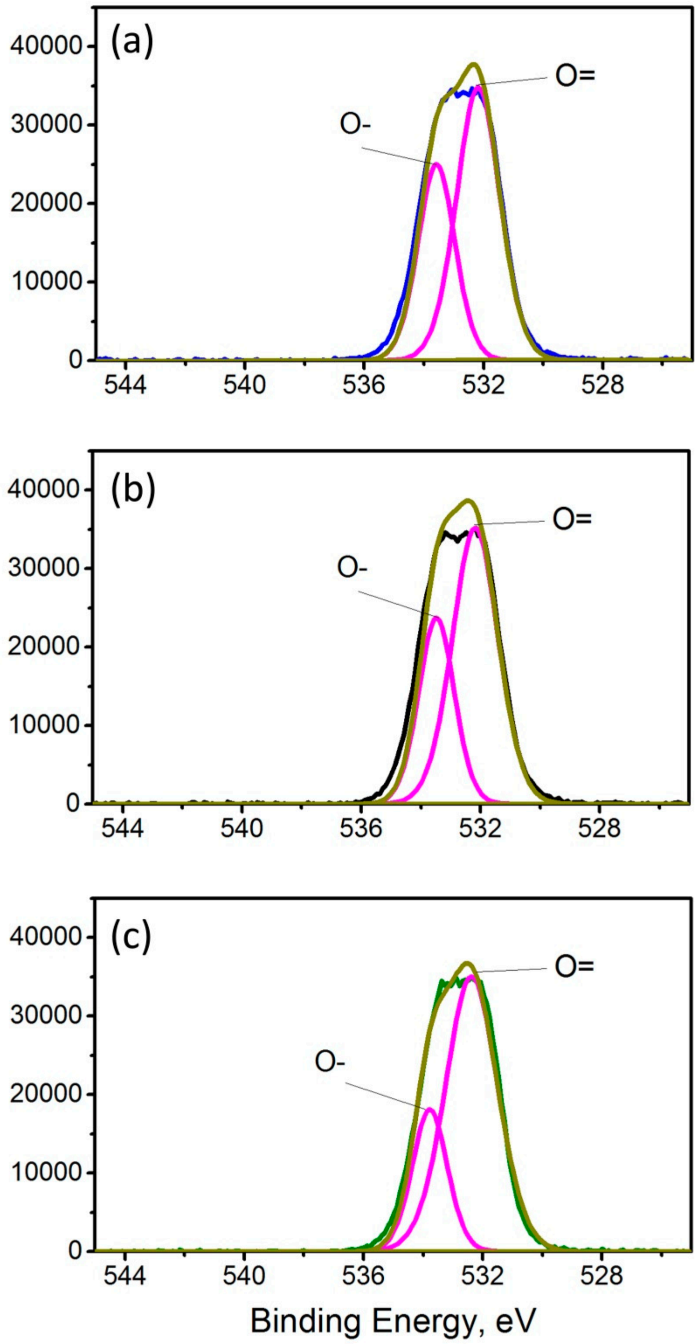


**Figure S4** (a-c) High-resolution O1s XPS core-level spectra of the MIPs-1 (a), MIPs-2 (b) and MIPs-3 (c) samples.

In the present study, basic yellow 1 (BY), methylene blue (MB) and safranine T (ST) were used as the competing molecules against malachite green (MG). After the aqueous mixture of the mixed dyes was processed by the adsorbents, the residual concentrations of the different kinds of dyes were tested by an ion trap mass spectrometer, and the adsorption capacities of the various dyes onto the microspheres were also calculated. Before the mixture of dyes was prepared, the standard mass spectrum of the each neat dye was analyzed, in order to the avoid the overlap of the ion peaks. The standard mass spectrum of the mixed dye solution is provided in Figure S1. The ion peaks at 283.1, 284.2, 315.1 and 329.2 *m*/*z* can be assigned to MG, BY, MB and ST, respectively. A series of mixed solution with different concentrations were prepared in

order to produce the internal standard curve, and deuterated MG was employed as the internal standard (334.2 *m*/*z*). The resulting linear correlation coefficients are presented in Table S1 in the ESI.

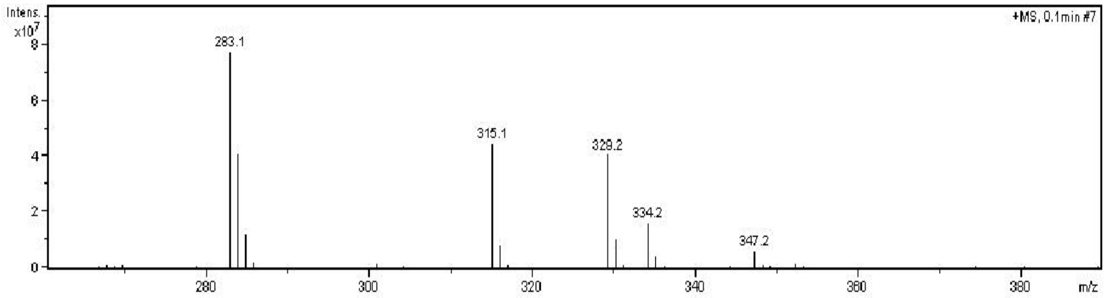


**Figure S5** Mass spectrum of various competing molecules in the mixed dye solution.

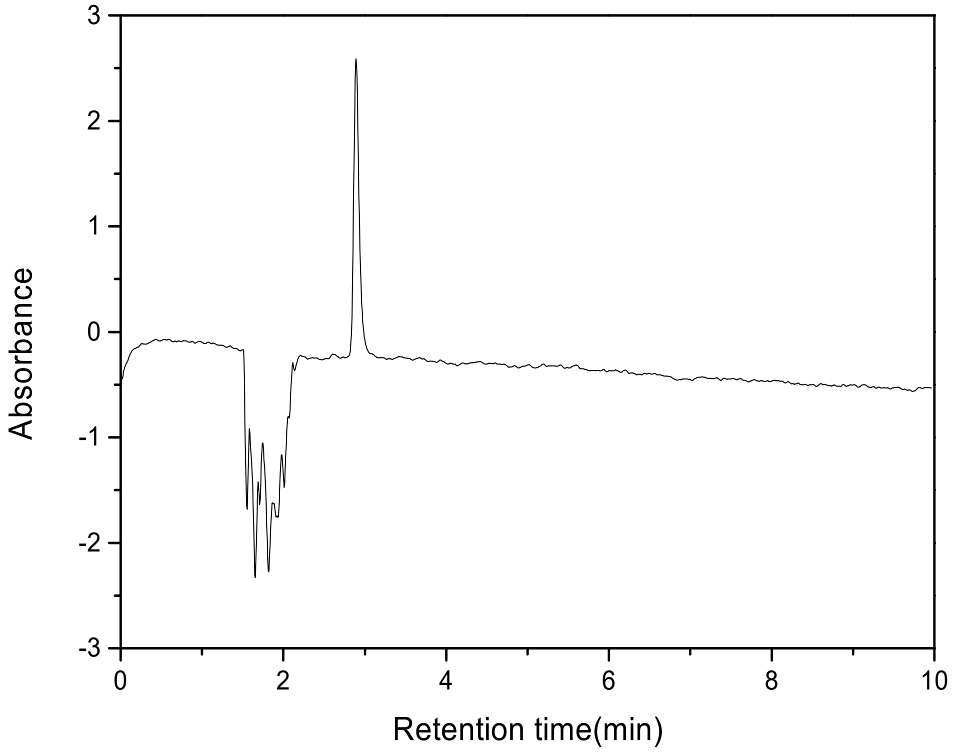


**Figure S6** Standard Chromatogram of a MG solution (0.2 mg/L).

**Table S1** Summary of the results from the $N_2$ adsorption-desorption measurements for the prepared NIPs, MIPs-1, MIPs-2 and MIPs-3 samples.

| Sample | BET surface area ($m^2$/g) | Pore volume (%) | | | Total pore volume ($cm^3$/g) |
|---|---|---|---|---|---|
| | | Micropore (<2 nm) | Mesopore (2-50 nm) | Macropore (>50 nm) | |
| NIPs | 9.250 | 0 | 86.4 | 13.6 | $1.670\times10^{-2}$ |
| MIPs-1 | 9.786 | 0 | 86.6 | 13.4 | $1.783\times10^{-2}$ |
| MIPs-2 | 3.396 | 0 | 69.4 | 30.6 | $8.358\times10^{-3}$ |
| MIPs-3 | 5.975 | 0 | 81.8 | 18.2 | $1.090\times10^{-2}$ |

**Table S2** Linear correlation coefficients obtained for the competing molecules involved in this study.

| Competing molecules | Ion peak position | Regression equation | Linear correlation coefficient ($R^2$) |
|---|---|---|---|
| BY | 283 | $y = 0.5858x + 0.161$ | 0.9909 |
| MB | 284 | $y = 0.5549x + 0.0622$ | 0.9892 |
| ST | 315 | $y = 0.3763x + 0.1691$ | 0.9918 |
| MG | 329 | $y = 0.3773x + 0.0156$ | 0.9998 |

## Reference


1. Bergaoui, M.; Khalfaoui, M.; Villarroel-Rocha, J.; Barrera, D.; Al-Muhtaseb, S.; Enciso, E.; Sapag, K.; Ben Lamine, A., New insights on estimating pore size distribution of latex particles: Statistical mechanics approach and modeling. *Microporous Mesoporous Mater.* **2016,** *224*, 360-371.
2. Johari, I.; Yusof, N.; Haron, M.; Nor, S., Preparation and Characterization of Poly(ethyl hydrazide) Grafted Oil Palm Empty Fruit Bunch for Removal of Ni(II) Ion in Aqueous Environment. *Polymers* **2013,** *5* (3), 1056-1067.
3. Roh, H.-K.; Kim, H.-K.; Kim, M.-S.; Kim, D.-H.; Chung, K. Y.; Roh, K. C.; Kim, K.-B., In situ synthesis of chemically bonded NaTi2(PO4)3/rGO 2D nanocomposite for high-rate sodium-ion batteries. *Nano Res.* **2016,** *9* (6), 1844-1855.
